# Effect of educational intervention based on protection motivation theory on preventive behaviors of respiratory infections among hospital staff

**DOI:** 10.3389/fpubh.2023.1326760

**Published:** 2024-01-05

**Authors:** Tayebeh Rakhshani, Sepahdar Nikeghbal, Seyyed Mansour Kashfi, Amirhossein Kamyab, Pooyan Afzali Harsini, Ali Khani Jeihooni

**Affiliations:** ^1^Nutrition Research Center, Department of Public Health, School of Health, Shiraz University of Medical Sciences, Shiraz, Iran; ^2^Department of Public Health, School of Health, Shiraz University of Medical Sciences, Shiraz, Iran; ^3^Department of Public Health, School of Health, Shiraz University of Medical Sciences, Shiraz, Iran; ^4^Department of Community Medicine, School of Medicine, Fasa University of Medical Sciences, Fasa, Iran; ^5^Department of Public Health, School of Health, Kermanshah University of Medical Sciences, Kermanshah, Iran; ^6^Nutrition Research Center, Department of Public Health, School of Health, Shiraz University of Medical Sciences, Shiraz, Iran

**Keywords:** educational intervention, preventive behaviors, respiratory infections, respiratory infections, protection motivation theory

## Abstract

**Background:**

Hospital staff represent a vulnerable population for respiratory diseases. Consequently, the implementation of training programs becomes imperative as a preventive measure against such infections in these populations. The current study was conducted to examine the impact of an educational intervention based on the Protection Motivation Theory (PMT) on preventive behaviors for respiratory infections among a group of hospital staff.

**Methods:**

This experimental study involves a sample of 150 hospital staff from Gachsaran City, Iran, in 2021–2022. The sampling technique involved the utilization of a random assignment approach to allocate individuals into two distinct groups: the experimental group, consisting of 75 participants, and the control group, also including 75 individuals. The data collection instrument was a questionnaire designed in accordance with the PMT. This questionnaire was administered to both the experimental and control groups prior to the intervention as well as two months following the intervention. The intervention program consisted of a total of five sessions, each lasting for 60 min, for the experimental group. These sessions were conducted on a weekly basis over a period of two and a half months. Specifically, there were two sessions held every month and one session held every two weeks. Following the completion of the program, the data was entered into SPSS-24 statistical software for analysis using paired t-tests, independent t-tests, and chi-square tests.

**Results:**

The results indicated that prior to the intervention, there was no significant difference between the two groups in terms of perceived vulnerability constructs (*p* = 0.25), perceived severity (*p* = 0.63), perceived response (*p* = 0.32), and perceived reward (*p* = 0.11). Besides, there was no considerable distinction in perceived self-efficacy (*p* = 0.84), perceived response cost (*p* = 0.33), fear (*p* = 0.45), behavior motivation (*p* = 0.51), knowledge (*p* = 92), or vaccination behavior (*p* = 0.12) before the educational intervention. However, a significant change was noticed in each of the mentioned variables between the two groups after the intervention (*p* < 0.05).

**Conclusion:**

The results of this study indicated that the implementation of an educational intervention grounded in the PMT yields positive outcomes in enhancing preventative behaviors pertaining to respiratory infections. Hence, it is recommended to utilize an intervention grounded in this theory among hospital staff as a viable approach to mitigating the occurrence of respiratory infections.

## Background

Acute respiratory tract infections are prevalent and can lead to significant and unfavorable health outcomes for patients (1–3). This category of infections encompasses a diverse array of illnesses, including common colds, throat infections, tonsillitis, influenza, COVID-19, and lower respiratory tract diseases (4). The etiological agents responsible for these diseases can be viral, bacterial, fungal, or even parasitic in nature (5–7). This particular ailment is responsible for around one-third of fatalities that occur within hospital settings, while also extending the length of hospital stays and incurring additional expenses related to treatment (8). Consequently, the failure of hospital staff to adhere to infection control protocols was identified as a mental health issue (9, 10). In 2019, a highly perilous respiratory infectious disease emerged and rapidly attained global prevalence, resulting in several fatalities (11). On January 30, 2020, the World Health Organization officially declared the dissemination of this disease the sixth leading cause of a global public health emergency. The causative agent was identified as the coronavirus (12–14).

In contrast to other administrative and service businesses, hospitals and medical facilities pose a higher level of risk to their workers (15). One of the most prevalent occupational risks faced by healthcare workers is the potential exposure to biological agents, which is subsequently associated with the risk of infection (15). Healthcare professionals are at the forefront of combating infectious diseases and thus face a higher risk of infection, making them more susceptible to the consequences associated with such disorders (16). The research findings indicate that there was an 8.3% rate of infection among medical care workers during the COVID-19 outbreak. This high percentage of infections can be attributed to the lack of protective measures taken by medical workers during their initial contact with infected patients at the onset of the outbreak (17).

The implementation of preventive measures by healthcare professionals to safeguard their own well-being against respiratory infections is a pivotal component of the respiratory infection prevention and control program (18). One of the crucial and indispensable techniques for the prevention and control of respiratory infections is the implementation of preventive measures, which encompass educational initiatives, the enhancement of public knowledge, and the development of personal protective skills (19). The initial stage in the planning process of a health education program involves the selection of an appropriate model. This model serves as a guiding framework, ensuring that the program remains focused and aligned with its intended objectives (19).

The Protection Motivation Theory (PMT) is a prominent educational framework that has been proposed in the field of health education (20). This idea was posited by Rogers in 1975 as a means to elucidate the impact of fear on attitudes and behaviors pertaining to health (21). This approach posits that the adoption of health behaviors aimed at mitigating health risks is directly influenced by an individual’s incentive to safeguard their own well-being (22). Rogers suggested that fear affects protection motivation (or the intention to perform protective measures against risks) through five constructs, and ultimately protection motivation causes healthy behaviors, these five constructs are: perceived vulnerability (a person’s belief that they are vulnerable to a health hazard), perceived severity (a person’s belief that the hazard is serious), perceived response efficacy (a person’s expectation that an adaptive response can eliminate the hazard), perceived response costs (a person’s estimate of any costs, such as money, people, time, and effort, associated with the protected behavior), and perceived self-efficacy (a person’s belief that they can successfully perform the behavior) (22).

In some studies, the effectiveness of health interventions based on the PMT has been mentioned in preventing various diseases; for example, Kowalski et al. (23), Ekow Arkorful et al. (24), and Salmani et al. (25) have stated that high blood pressure can be controlled by using health interventions (23–25). Healthcare professionals maintain a direct association with both individuals afflicted by respiratory infections and those who are in good health. Consequently, it is imperative to prioritize the well-being of these employees, as it serves the dual purpose of safeguarding them against respiratory infections and curbing the transmission of such infections within the wider community. It is vital to identify the preventative measures and control strategies for respiratory infections, as well as the determinants and factors that influence individuals’ adoption of preventive behaviors in relation to such infections.

Given the imperative nature of engaging in protective measures against respiratory infections and adhering strictly to health protocols within the healthcare profession, it is crucial to examine the attitudes and beliefs surrounding the rigorous implementation of these behaviors among healthcare workers. This analysis will inform the development and execution of appropriate educational interventions aimed at fostering a culture of adherence and promoting these protective behaviors. The adoption of protective behaviors is expected to yield positive outcomes. Given their role as guardians of the health and well-being of other individuals within society, it is imperative to assess the health behaviors of these individuals based on the outcomes observed. Consequently, it is crucial to undertake measures aimed at preserving and enhancing their own health. Hence, the current study was devised and executed with the objective of assessing the impact of an educational intervention rooted in the PMT on the adoption of preventive behaviors against respiratory diseases among the hospital staff in Gachsaran city, Iran.

## Methods

### Study design and participants

This experimental study was conducted in 2021–2022 among the employees of Gachsaran City Hospital. The criteria for entering the study were having work experience of more than one year at the hospital and having contact with infectious diseases at the time of the study. Exclusion criteria were not wanting to cooperate at any time during the study, changing the workplace during the implementation of the study, and not participating in more than two training sessions.

Considering the mean comparison formula in two communities and also according to the results of similar research by Bashirian et al. (26), the sample size was determined to be 150 people using the census method (26).


$$n=\frac{{\left(Z_{1-\frac{\alpha }{2}}+Z_{1-\beta }\right)}^{2}\left(\delta_{1}^{2}+\delta_{2}^{2}\right)}{{\left(\mu_{1}-\mu_{2}\right)}^{2}}$$

### Data collection tools

A demographic characteristics questionnaire was initially completed, including age, sex, marital status, education, related ward, work experience, and monthly income.

Then, the PMT questionnaire was completed. It included three parts: knowledge and behavior, PMT constructs, and vaccination behavior. Knowledge questions included 15 questions based on a 5-point Likert scale (strongly agree, agree, no opinion, disagree, and strongly disagree) regarding knowledge of preventive behaviors against respiratory infections. Behavior questions included nine questions based on a 5-point Likert scale (strongly agree, agree, no opinion, disagree, and strongly disagree) regarding the preventive behaviors of respiratory infections.

In the PMT constructs section, perceived sensitivity, perceived severity, perceived response cost, perceived reward, and perceived self-efficacy were assessed. Perceived sensitivity was evaluated using four questions based on a 5-point Likert scale (strongly agree, agree, no opinion, disagree, and strongly disagree) regarding the subjects’ perceptions of the preventive behaviors of respiratory infections. The lowest and highest scores were 8 and 40, respectively.

Perceived severity was evaluated using seven questions based on a 5-point Likert scale (strongly agree, agree, no opinion, disagree, and strongly disagree) regarding the subjects’ perceptions of the preventive behaviors of respiratory infections. The lowest and highest scores were 6 and 25, respectively. Perceived response cost was evaluated using six questions based on a 5-point Likert scale (strongly agree, agree, no opinion, disagree, and strongly disagree) regarding the subjects’ perceptions of the preventive behaviors of respiratory infections. The lowest and highest scores were 5 and 30, respectively.

Perceived reward was evaluated using five questions based on a 5-point Likert scale (strongly agree, agree, no opinion, disagree, and strongly disagree) regarding the subjects’ perceptions of the preventive behaviors of respiratory infections. The lowest and highest scores were 6 and 25, respectively. Perceived self-efficacy was evaluated using 10 questions based on a 5-point Likert scale (strongly agree, agree, no opinion, disagree, and strongly disagree) regarding the subjects’ ability to perform preventive behaviors for respiratory infections. The lowest and highest scores were 3 and 15, respectively.

Lastly, the vaccination questionnaire was designed based on similar studies, including Salimi et al. (27), Kashmiri et al. (28), Rahimi et al. (29), and Fakharian Moghadam et al. (30). This part included 17 questions based on a 5-point Likert scale (strongly agree, agree, no opinion, disagree, strongly disagree) regarding the subjects’ perception of vaccination. The lowest and highest scores were 7 and 40, respectively.

The questionnaire was based on a study by Bashirian et al., whose validity and reliability were confirmed (26). In the present study, content validity was confirmed using the opinions of 10 health education and promotion specialists, and internal consistency methods were used to measure the reliability of the tool. Using SPSS version 24, the Cronbach’s alpha coefficient was determined for the entire questionnaire. Cronbach’s alpha coefficient for perceived sensitivity, severity, self-efficacy, response cost, and behavior motivation was measured as 0.86, 0.88, 0.86, 0.85, 0.83, and 0.86, respectively. The coefficient for the entire questionnaire was measured at 0.87.

### Procedure

Upon receipt of the code of ethics from the ethics commission, the researchers commenced their job, and individuals who met the criteria for participation in the study were selected. The researchers provided a detailed explanation of the project’s objectives to the prospective participants, who expressed their interest in taking part. Subsequently, these individuals were admitted into the study upon completion of their written informed consent. Following this, the participants were administered questionnaires, which were subsequently completed by the selected sample population. The individual in question established the designation for the intervention cohort after revisiting the hospital to extend invitations to the members of said cohort. During this encounter, the individual provided a comprehensive explanation about the training sessions, their objectives, and the designated location for the meetings. Furthermore, the individuals were cordially invited to actively partake in these gatherings. The researchers subsequently facilitated the scheduling of sessions in collaboration with the retraining unit within the aforementioned center, with the assistance of a health expert and a health promotion expert. Following the educational intervention, the questionnaires were gathered after a period of two months.

### Educational intervention

To design the intervention, first a PMT questionnaire was completed by the participants. Then, after finding the weaknesses and strengths of the participants, the educational design was developed based on their weak points. Finally, the intervention included five 60-min sessions and was considered in the form of teaching methods (lectures, questions and answers, group discussions, PowerPoint presentations, pamphlets, and video clips) in the retraining center of health programs in Gachsaran City under the supervision of the health network to instruct behaviors to prevent respiratory infections in the intervention group. Two months after the intervention, the data was collected again and compared with before the intervention. During intervention, an expert group, including a health expert and a health promotion expert, collaborated; each of them specialized in training items related to the PMT.

**Table:** 

	Training method	Duration	Subject	Purpose	Trainer
1^st^ session	Lecture and Q&A	60	Statement of purpose, introducing the participants	General purpose	Researcher
2^nd^ session	PowerPoint	60	Introducing the disease with the aim of increasing subjects’ knowledge	Introducing infectious diseases	Health specialist
3^rd^ session	Video clip	60	Introducing the model and its constructs	Introducing PMT	Health promotion expert and researcher
4^th^ session	Video clip	60	Importance of protection and prevention of respiratory infections	Model-based training	Health promotion expert and researcher
5^th^ session	Group discussion	60	Review and final evaluation	Review and summary	Researcher

### Data analysis

The data was analyzed by SPSS version 24 statistical software, so that the normality of the data was first measured through the Kolmogorov–Smirnov test. Frequency, mean, and standard deviation indexes were used to describe the data, and independent t-test, chi-square test, and paired t-test were used to compare the average data in the two groups before and after the intervention. The significance level was considered to be 0.05 in all tests.

## Results

Table 1 shows the demographic characteristics of the hospital staff participating in the study. The mean and standard deviation of the subjects’ ages in the experimental and control groups were 38.19 ± 3.01 and 37.88 ± 4.12 years, respectively. The mean and standard deviation of work experience in the experimental and control groups were 7.14 ± 3.51 and 7.33 ± 4.12 years, respectively. Based on the independent t-test, there was no significant difference between the two groups in terms of age (*p* = 0.45) or work experience (*p* = 0.66). Likewise, based on the results of the chi square test, there was no significant difference between the experimental and control groups in terms of gender (*p* = 0.54), marital status (*p* = 0.18), education (*p* = 0.11), related ward (*p* = 0.35), and monthly income (*p* = 0.16) (Table 1).

**Table 1:** Frequency distribution of primary variables of the study participants.

Variable		Intervention (%)	Control (%)	*p* value
Gender	Male	45 (60)	40 (53.33)	0.54
	Female	30 (40)	35 (46.66)	
Marital status	Single	20 (26.66)	18 (24)	0.18
	Married	45 (60)	50 (66.66)	
	Other	10 (13.3)	7 (9.33)	
Education	High school	1 (1.33)	2 (2.66)	0.11
	College	2 (2.66)	3 (4)	
	University	72 (96)	70 (93.34)	
Related ward	Health staff	40 (53.33)	50 (66.66)	0.35
	Utility	3 (4)	5 (6.66)	
	Other	32 (42.66)	20 (26.66)	
Monthly income	100–150 million IR Rials	5 (6.66)	4 (5.33)	0.16
	>150 million IR Rials	70 (93.34)	70 (93.34)	

Table 2 shows the subjects’ knowledge of respiratory infections. Most of the participants in the two groups (96% of the experimental group and 93.33% of the control group) had received training about respiratory infections, and most of them (97.33% of the experimental group and 96% of the control group) had received the COVID-19 vaccine. Most of the participants (60% of the experimental group and 66.66% of the control group) mentioned the Internet as their main source of information.

**Table 2:** Frequency distribution of the participants’ knowledge about respiratory infections.

Variable		Intervention (%)	Control (%)	*p* value
Training related to respiratory infections	Yes	72 (96)	70 (93.33)	0.44
	No	3 (4)	5 (6.67)	
Vaccination	Yes	73 (97.33)	72 (96)	0.93
	No	2 (2.66)	3 (4)	
Being infected with respiratory infections	Yes	70 (93.33)	71 (94.66)	0.25
	No	5 (6.67)	4 (5.33)	
Getting info on respiratory infections	Social networks	45 (60)	50 (66.66)	0.16
	Hoardings	15 (20)	10 (13.33)	
	Pamphlets	0	2 (2.66)	
	Health staff	5 (6.66)	7 (9.33)	
	Other	10 (13.33)	8 (10.66)	

Table 3 shows the mean and standard deviation of the PMT constructs before and after the intervention in the two experimental and control groups. Based on the results, there was no significant difference between the two groups before the intervention in terms of perceived sensitivity (*p* = 0.25), perceived severity (*p* = 0.63), perceived response (*p* = 0.32), perceived reward (*p* = 0.11), perceived self-efficacy (*p* = 0.84), perceived response cost (*p* = 0.33), fear (*p* = 0.45), behavior motivation (*p* = 0.51), knowledge (*p* = 0.92), behavior (*p* = 0.12), and vaccination behavior (*p* = 0.35). However, after the intervention, there was a significant difference between the two groups in terms of perceived sensitivity, perceived severity, perceived response, perceived reward, perceived self-efficacy, perceived response cost, fear, behavior motivation, knowledge, behavior, and vaccination behavior (*p* < 0.001).

**Table 3:** Mean and standard deviation of PMT constructs before and after the intervention.

Constructs	Group	Before intervention (M ± SD)	After intervention (M ± SD)	*p* value
Perceived sensitivity	Intervention	12.51 ± 21.5	16.64 ± 2.36	0.001
	Control	11.45 ± 1.13	10.35 ± 2.51	0.84
	value of p	0.25	0.001	
Perceived severity	Intervention	18.71 ± 3.84	24.72 ± 3.79	0.001
	Control	18.54 ± 2.94	18.65 ± 1.84	0.65
	value of p	0.63	0.001	
Perceived response	Intervention	25.84 ± 3.95	29.90 ± 2.46	0.001
	Control	25.76 ± 3.83	24.74 ± 2.33	0.36
	value of p	0.32	0.001	
Perceived reward	Intervention	11.36 ± 3.44	15.43 ± 5.76	0.001
	Control	10.26 ± 3.21	10.45 ± 2.10	0.92
	value of p	0.11	0.001	
Perceived self-efficacy	Intervention	20.32 ± 6.85	40.20 ± 1.48	0.001
	Control	20.65 ± 5.86	19.45 ± 4.91	0.11
	value of p	0.84	0.001	
Perceived response cost	Intervention	15.31 ± 4.69	19.29 ± 6.85	0.001
	Control	14.33 ± 3.99	14.29 ± 45.2	0.45
	value of p	0.33	0.001	
Fear	Intervention	8.76 ± 1.68	10.90 ± 2.02	0.001
	Control	7.66 ± 2.31	6.33 ± 2.02	0.67
	value of p	0.45	0.001	
Behavior motivation	Intervention	15.41 ± 2.31	22.92 ± 3.48	0.001
	Control	15.64 ± 2.54	15.15 ± 2.86	0.62
	value of p	0.51	0.001	
Behavior	Intervention	20.24 ± 3.95	30.75 ± 2.83	0.001
	Control	21.28 ± 2.95	20.19 ± 2.45	0.32
	value of p	0.12	0.001	
Knowledge	Intervention	50.18 ± 3.64	59.16 ± 5.15	0.001
	Control	51.18 ± 3.95	50.65 ± 3.45	0.87
	value of p	0.92	0.001	
Vaccination behavior	Intervention	50.98 ± 6.33	70.85 ± 5.64	0.001
	Control	60.97 ± 5.65	60.48 ± 4.66	0.52
	value of p	0.35	0.001	

## Discussion

The present study was conducted with the aim of determining the effect of an educational intervention based on the PMT on the preventive behaviors of respiratory infections in a group of hospital staff in Gachsaran City. The results of the present study showed that, after the educational intervention, there was a significant difference between the experimental and control groups in terms of perceived sensitivity. The mean of this construct in the experimental group was higher than in the control group, which could somehow be attributed to the training. In justification of this, in addition to the training given based on the PMT, due to the coincidence of the study with the pandemic, the participants became more sensitive to infectious diseases and took more preventive measures against infectious diseases. This finding was consistent with the results of studies by Nguyen et al. (31)and Ryu et al. (32).

The results of the present study showed that after the intervention, there was a significant difference between the experimental and control groups in terms of perceived severity. The mean of this construct in the experimental group was higher than the control group. One of the possible reasons for this issue is that when people feel vulnerable about something, they try hard to refrain from doing it; the same is the case with infectious diseases (33). In this way, when a person understands his vulnerability, he tries to prevent the disease by observing a series of health principles. These results were consistent with the results of studies by Byrd et al. (34) and Khaday et al. (35).

The results of the present study showed that after the educational intervention, there was a significant difference between the experimental and control groups in terms of perceived response. The mean of this construct in the experimental group was higher than the control group, which is consistent with the results of studies by Grano et al. (36) aimed at the application of PMT in COVID-19 (2022), and Nawabi et al. (37).

Our results showed that after the intervention, there was a significant difference between the experimental and control groups in terms of perceived reward. The mean of this construct in the experimental group was higher than the control group. In justification of this finding, it can be stated that training based on the PMT can make the learner aware of the reward they receive as a result of protection; in other words, they accept that there is a reward in doing so. Regarding infectious diseases, hospital staff are well aware that by observing health and protection issues, there is safety against infectious diseases. This could be the reward itself. These findings are in agreement with the results of studies by Hedayati et al. (38) and Elgzar et al. (39).

Based on our results, after the intervention, there was a significant difference between the experimental and control groups in terms of perceived self-efficacy. The mean of this construct in the experimental group was higher than the control group. When a person does not have the necessary self-efficacy, they cannot observe their preventive behaviors and are exposed to respiratory infections (40). Consistently, in a meta-analysis by Zaildo et al. (41), they stated many obstacles and benefits for preventing respiratory infections (41).

According to the findings, after the educational intervention, there was a significant difference between the experimental and control groups in terms of perceived response cost. It can be said that perceived response cost is an important factor in preventing respiratory infections. It is natural that people seek to reduce unnecessary costs, and preventing respiratory infections can be an unwanted cost. These findings are in line with the results of studies by Calcagni et al. (42) and Lapoirie et al. (43).

The results of the present study showed that after the educational intervention, there was a significant difference between the experimental and control groups in terms of behavior motivation. For the possible justification of this finding, it can be stated that motivation is an important factor in performing a healthy behavior, and the main factor in performing a healthy behavior or leaving an unhealthy behavior is actually motivation. Consistently, the results of studies by Yoon et al. (44), Acar et al. (45), Meng et al. (46), and Leung et al. (47) reported similar findings in improving behavior in their studied groups (44–47).

Our results noted that after the educational intervention, there was a significant difference between the experimental and control groups in terms of fear, and the mean of this construct was higher in the experimental group than the control group, which is consistent with the results of studies by Howell et al. (48), Downing et al. (49), and Hodge et al. (50).

Our findings indicated that after the educational intervention, there was a significant difference between the experimental and control groups in terms of knowledge. It can be stated that the nature of education is that it makes a person aware of the subject that is taught to him; on the other hand, hospital staff have passed training courses on respiratory diseases and are fully aware of these diseases. The influence of education on knowledge and knowledge is similarly reported in the studies by Unger et al. (36), Grano et al. (51), and Abdel-Aziz et al. (52).

Finally, the outcomes of the present study highlighted that after the educational intervention, there was a significant difference between the experimental and control groups in terms of vaccination behavior. The present study coincided with COVID-19, when the only way to prevent the disease was to receive a vaccine. Therefore, the hospital staff, through the training provided by the researcher and other means, were fully aware of receiving the vaccine. Also, since vaccination was mandatory, the employees were forced to receive it. The efficacy of education on vaccination behavior was similarly reported in the studies by Hadizadeh et al. (6), Prince et al. (53), and Wu et al. (54).

### Strengths and limitations

The study exhibits several notable strengths. Firstly, it demonstrates active engagement from the hospital staff in the study’s execution. Secondly, the questionnaire employed encompasses all relevant questions pertaining to the idea of protection. Moreover, the education initially targeted the weak points of the participants for more efficacy. Lastly, it is worth mentioning that the researcher was present throughout all stages of data collection.

The short-term evaluation of the impact of the educational program and the data collection via questionnaires, which were collected using a self-reporting method, were subject to certain limitations. One limitation relates to the potential inaccuracies and lack of authenticity in the information provided by some participants. Additionally, the specific cultural context present in Gachsaran City limited the generalizability of the findings to other places.

## Conclusion

The results of the present study showed that the use of PMT was able to lead to a change in constructs such as perceived sensitivity, perceived severity, knowledge, and behavior motivation in employees toward respiratory diseases, so it can be suggested that educational interventions aimed at applying the PMT be implemented in the case of other diseases. In the same way, it is possible to teach compliance with health issues using this model in staff retraining courses.

## Data availability statement

The data that support the findings of this study are available from the corresponding author upon reasonable request.

## Ethics statement

The studies involving humans were approved by the Human Research Ethics Committee at the Shiraz university of medical sciences (IRSUMS.SCHEANUT.REC.1400.077). The studies were conducted in accordance with the local legislation and institutional requirements. The participants provided their written informed consent to participate in this study.

## Author contributions

TR: Conceptualization, Investigation, Methodology, Project administration, Supervision, Visualization, Writing – review & editing. SN: Conceptualization, Methodology, Writing – review & editing, Data curation, Formal analysis, Software, Writing – original draft. SK: Conceptualization, Methodology, Writing – original draft, Project administration, Supervision, Visualization. AK: Conceptualization, Supervision, Visualization, Investigation, Resources, Software, Validation, Writing – review & editing. PH: Conceptualization, Investigation, Resources, Software, Supervision, Validation, Visualization, Writing – review & editing, Data curation, Formal analysis, Methodology. AJ: Conceptualization, Data curation, Formal analysis, Investigation, Methodology, Resources, Supervision, Validation, Visualization, Writing – review & editing, Project administration, Writing – original draft.
